# Neurodegeneration: can metabolites from *Eremurus persicus* help?

**DOI:** 10.3389/fphar.2024.1309766

**Published:** 2024-02-05

**Authors:** Valeria Cavalloro, Nicoletta Marchesi, Pasquale Linciano, Daniela Rossi, Lucrezia Irene Maria Campagnoli, Alice Fossati, Karzan Mahmood Ahmed, Alessio Malacrida, Mariarosaria Miloso, Giuseppe Mazzeo, Sergio Abbate, Giovanna Longhi, Francesca Alessandra Ambrosio, Giosuè Costa, Stefano Alcaro, Alessia Pascale, Emanuela Martino, Simona Collina

**Affiliations:** ^1^ Department of Earth and Environmental Sciences, University of Pavia, Pavia, Italy; ^2^ National Biodiversity Future Center, Palermo, Italy; ^3^ Department of Drug Sciences, University of Pavia, Pavia, Italy; ^4^ Department of Chemistry, College of Education, University of Garmian, Kalar, Iraq; ^5^ School of Medicine and Surgery, University of Milan-Bicocca, Monza, Italy; ^6^ Experimental Neurology Unit, University of Milano-Bicocca, Monza, Italy; ^7^ Department of Molecular and Translational Medicine, Università di Brescia, Brescia, Italy; ^8^ Department of Experimental and Clinical Medicine, University “Magna Græcia” of Catanzaro, Campus “S. Venuta”, Catanzaro, Italy; ^9^ Department of Health Sciences, Campus “S. Venuta”, “Magna Græcia” University of Catanzaro, Catanzaro, Italy; ^10^ Net4Science Academic Spin-Off, University “Magna Græcia” of Catanzaro, Campus “S. Venuta”, Catanzaro, Italy; ^11^ Associazione CRISEA–Centro di Ricerca e Servizi Avanzati per l’Innovazione Rurale, Italy

**Keywords:** *Eremurus persicus*, HuD, embryonic lethal abnormal visual-like, proteasome activators, molecular modeling, *(R)*-aloesaponol-III-8-methyl ether, *(R)*-germichrysone, nature-aided drug discovery

## Abstract

The number of patients affected by neurodegenerative diseases is increasing worldwide, and no effective treatments have been developed yet. Although precision medicine could represent a powerful tool, it remains a challenge due to the high variability among patients. To identify molecules acting with innovative mechanisms of action, we performed a computational investigation using SAFAN technology, focusing specifically on HuD. This target belongs to the human embryonic lethal abnormal visual-like (ELAV) proteins and plays a key role in neuronal plasticity and differentiation. The results highlighted that the molecule able to bind the selected target was (*R*)-aloesaponol-III-8-methyl ether [(*R*)-ASME], a metabolite extracted from *Eremurus persicus.* Notably, this molecule is a TNF-α inhibitor, a cytokine involved in neuroinflammation. To obtain a suitable amount of (*R*)-ASME to confirm its activity on HuD, we optimized the extraction procedure. Together with ASME, another related metabolite, germichrysone, was isolated. Both ASME and germichrysone underwent biological investigation, but only ASME confirmed its ability to bind HuD. Given the multifactorial nature of neurodegenerative diseases, we decided to investigate ASME as a proteasome activator, being molecules endowed with this kind of activity potentially able to counteract aggregations of dysregulated proteins. ASME was able to activate the considered target both in enzymatic and cellular assays. Therefore, ASME may be considered a promising hit in the fight against neurodegenerative diseases.

## 1 Introduction

In recent years, changes in the demographic trend have led to progressive aging of the population worldwide (Klimova and Kuca, 2016). This scenario is associated with a continuous increase in the number of patients affected by neurodegenerative diseases, among which the most widespread are Alzheimer’s, Parkinsonism, and amyotrophic lateral sclerosis diseases (Logroscino et al., 2022; Duarte Folle et al., 2023). The treatments available in this field are often disease-specific and involve the use of small molecules, like memantine, carbidopa, or riluzole, or monoclonal antibodies, like aducanumab, but they are always associated with poor efficacy (Lamptey et al., 2022). Despite many efforts in recent years, no effective treatments have been developed, and patients affected by these pathologies still have a poor quality of life (Maresova et al., 2020). In this scenario, precision medicine may be a powerful tool, but this approach is still challenging due to the high variability among patients. Given the multifactorial nature of neurodegenerative disorders, the development of multi-target drugs acting with innovative mechanisms could represent a winning strategy in developing novel effective treatments against such diseases (Kamecki et al., 2021; Pellavio et al., 2021; Linciano et al., 2023).

To identify molecules able to interact with targets potentially involved in neurodegenerative diseases and acting through multiple mechanisms of action, for this work, we used our in-house compound library. Such a collection, which includes both synthetic molecules and secondary metabolites, has been digitized to speed up the drug discovery process. Collected compounds are characterized by significant structural diversity, lead-likeness, and an overall solubility profile to ensure that the resulting hits are worthy of investigation (MedChem Lab, MCL, Department of Drug Sciences, University of Pavia, Pavia, Italy) (Listro et al., 2023; Pagano et al., 2023). Particularly, we focused on two targets, the RNA-binding protein named HuD and the proteasome system since proteasome activators are potentially able to counteract aggregations of dysregulated proteins (Leestemaker et al., 2017).

HuD protein belongs to the human embryonic lethal abnormal visual-like (ELAV) or Hu protein family, the main function of which is to increase the stability and/or the rate of translation of specific mRNAs. Four human ELAV proteins have been characterized: HuR (or HuA), HuB, HuC, and HuD, all sharing a high degree of sequence homology (70%–85%) (Volpe et al., 2020). Due to their ability to act in different contexts, ELAV proteins are considered pleiotropic proteins; however, while HuR is ubiquitous, HuB, HuC, and HuD are specifically expressed in the nervous system, and they are known as neuronal ELAV (nELAV; Vasile et al., 2018; Nishisaka et al., 2023). In particular, HuD, the most studied among the nELAV, has been recognized as a target dysregulated in several neurodegenerative disorders, like Alzheimer’s, Parkinsonism, and amyotrophic lateral sclerosis diseases, although only a few small molecules have been identified as binders so far (Ambrosio et al., 2021; Silvestri et al., 2022; Marchesi et al., 2023; Zhang et al., 2023).

Proteasomes are gaining more and more attention in the scientific community as they are responsible for maintaining cellular homeostasis under physiological conditions and modulating protein degradation during the life of the cell. While proteasome inhibitors as anticancer agents are deeply studied and are already in clinical practice, few data are available on proteasome activators. Recent evidence highlighted how enhancing proteasome activity can be an innovative tool in contrasting dysregulated protein aggregations in neurodegenerative disorders. Again, only a few small organic molecules endowed with this kind of activity have been identified, all being active in the mM range and with different mechanisms of action. Specifically, they have been described to act as inhibitors of deubiquitinase or p38 MAPK enzymes, modulators of cAMP- or cGMP-dependent protein kinase, or as direct 20S activators (e.g., SDS) (George and Tepe, 2021).

Due to the high novelty associated to these targets, the development of a new molecular entity able to both bind HuD and activate the proteasome has not been investigated yet. However, considering the multifactorial nature of neurodegenerative diseases, the development of new multi-target drugs could represent a pivotal step ahead in this field. More in detail, HuD and proteasome can be considered to be two orthogonal targets, one involved in differentiation and synaptic plasticity and the other in the degradation of proteins like synuclein or tau proteins.

In this paper, we report for the first time the medicinal chemistry work performed to identify novel dual ligands endowed with an innovative multi-target profile: HuD-RNA interfering compounds and proteasome activators, potentially active against neurodegenerative diseases.

## 2 Methods

### 2.1 SAFAN

SAFAN-*in silico* profiling (SAFAN-ISP) exploits a library of fragments and calculates similarity (Tanimoto) by matching atoms to fragments and evaluating common atoms. Experimental data are obtained using a refactored bioactivity database from the ChEMBL31 database. The steps performed by using the SAFAN platform foresee the fragmentation of the input molecule and the comparison of each fragment to the SAFAN-ISP database. Fragments are used to select targets sharing similar fragments with the input compounds and to calculate affinities by combining data concerning different compounds binding the same target. Next, the binding constant on all targets outputted is computed combining the similarities and experimental data using two schemes including chiral fingerprints and two excluding them. Finally, the RepTree algorithm, available from the WEKA open source package, is used to combine all binding constants obtained in the previous steps in a single value that will be the final output.

### 2.2 Molecular recognition studies

The (*R*)-aloesaponol-III-8-methyl ether [(*R*)-ASME] compound was prepared by means of the LigPrep tool. Hydrogens were added, salts were removed, and ionization states were calculated using an ionizer at pH 7.4 (SchrödingerLLC, 2018).

For the molecular modeling studies on the HuD protein, we started from the crystal structure of HuD and the (A/U)-rich element of c-fos mRNA, deposited in the Protein Data Bank (PDB) with the PDB code 1FXL (Wang and Tanaka Hall, 2001). The receptor structure was processed using the Protein Preparation Wizard tool (LLC, 2018) using the same protocol reported in our previous work (Ambrosio et al., 2021).

The docking studies were performed by means of the Glide v. 6.7 SP algorithm, and 10 poses per ligand were generated (Schrödinger, 2018).

For the modeling studies on the proteasome, we used the crystal structure of the human 20S proteasome complex with ixazomib, deposited in the PDB with the PDB code 5LF7 (Schrader et al., 2016). The structure was prepared by means of the Protein Preparation Wizard tool, using the same protocol reported in our previous works (Malacrida et al., 2021; Listro et al., 2022).

In order to identify and characterize the ligand-binding sites and druggability assessment in the *α*-rings of the 20S proteasome, the SiteMap program v. 4.6 (SiteMap, Schrödinger, 2018) was used.

According to the druggability score and SiteScore, the best SiteMap identified binding site was selected for further docking simulation, carried out with Glide software v. 6.7 by using the SP algorithm (Schrödinger, 2018), and 10 poses per ligand were generated.

### 2.3 Plant material and extraction procedure

*Eremurus persicus* (Jaub. & Spach) Boiss. was collected from a mountainous area (Küh-e Golestän, Golpayegan) located 120 km from Isfahan/Iran, at an altitude of 3000–3200 m. The collected plant materials were identified and classified by Dr. Abdulla Sa’ad at the Education Science Department, Faculty of Biology, Salahaddin University, Hawler/Iraq. The voucher specimen (No. 6856) was deposited at Education Salahaddin University Herbarium (ESUH), Hawler/Iraq. Freshly cut roots were dried in a drying room with active ventilation at room temperature (approximately 20°C–22°C) until they showed no further weight loss. The roots were cut into small pieces and ground with a blade mill (A10 IKA-Werke GmbH & Co. Staufen, Germany) to obtain a homogeneous fine powder. The plant material so treated was stored under dark conditions.

*E. persicus* extracts were prepared following both maceration and microwave-assisted solvent extraction (MASE) procedures. For the second method, a multi-mode microwave apparatus, using a closed-vessel system (MARSX press, CEM Corporation, Matthews, NC, United States), was exploited, setting 800 W as the maximum potency and 5 min as the ramp time. In order to identify the best extraction conditions in terms of (*R*)-ASME yields, the following conditions were applied (Table 1):

**TABLE 1:** Tested extraction conditions.

	# Cycles (time for each cycle)	T°C	Solvent renewal	Total EtOH volume (ml)
**Mac_1**	3 (1 h–12 h–1 h)	r.t.	Yes	600
**Mac_2**	3 (1 h–1 h–1 h)	r.t.	Yes	600
**Mac_3**	2 (12 h–1 h)	r.t.	Yes	400
**Mac_4**	2 (12 h–1 h)	60°C	Yes	400
**MASE_1**	1 (20 min)	120°C	Yes	200
**MASE_2**	1 (20 min)	60°C	Yes	200
**MASE_3**	2 (20 min + 20 min)	60°C	Yes	400
**MASE_4**	2 (5 min +5 min)	60°C	Yes	400
**MASE_5**	2 (20 min + 20 min)	60°C	No	200

After the extraction process, the solvent was evaporated under reduced pressure using a Heidolph Laborota 4000 instrument (Heidolph Instruments GmbH & Co., Schwabach, Germany) and the yield calculated (Table 2). Next, raw extracts were solubilized in water and extracted five times with ethyl acetate (5 × 80 ml). The organic phases so obtained were then washed with water (3 × 50 ml), dried with anhydrous Na_2_SO_4_, and evaporated under reduced pressure. This fraction was finally chromatographed (mobile phase: 1 hexane:1AcOEt; stationary phase: silica gel 60 particle size 230–400 mesh purchased from Nova Chimica, Cinisello Balsamo, Italy), allowing the isolation of pure (*R*)-ASME and an unknown metabolite. The entire process was followed exploiting analytical thin-layer chromatography (TLC) on silica gel pre-coated glass-backed plates (Fluka Kieselgel 60 F254, Merck, Darmstadt, Germany) using the same MP. The detection was conducted with UV light (254 and 366 nm).

**TABLE 2:** Extraction and pure metabolite yields related to each extraction protocol.

	Extraction yield (%)	(*R*)-ASME yield	((%)*R*)-Germichrysone yield
**M** (%)**ac_1**	13.8	0.14	0.011
**Mac_2**	9.1	0.11	0.033
**Mac_3**	12.9	0.21	0.051
**Mac_4**	25.8	0.24	0.062
**MASE_1**	20.1	0.14	0.044
**MASE_2**	5.9	0.16	0.060
**MASE_3**	8.5	0.24	0.081
**MASE_4**	9.0	0.27	0.075
**MASE_5**	6.6	0.18	0.028

(*R*)-ASME: [α]_D_ = −37.9 (*c* 0.04, CHCl_3_). ESI-MS: *m*/*z* 273 [M + H]^+^. ^1^H-NMR (CDCl_3_, 400 MHz): δ 2.01 (brs, 1H), 2.28 (m, 2H), 2.48 (s, 3H), 2.69 (m, 1H), 3.05 (m, 1H), 4.02 (s, 3H), 4.94 (dd, *J* = 5 Hz, 1H), 6.69 (d, *J* = 2 Hz, 1H), 7.06 (s, 1H), and 7.14 (d, *J* = 2 Hz, 1H).

^13^C-NMR (CDCl_3_): δ 203.3 (s), 165.9 (s), 159.5 (s), 142.0 (s), 140.3 (s), 139.9 (s), 119.9 (d), 115.4 (d), 113.8 (s), 109.2 (s), 108.3 (d), 68.0 (d), 56.0 (q), 34.0 (t), 30.7 (t), and 22.1 (q).

The structure of the unknown metabolite was then investigated with nuclear magnetic resonance (on a Bruker Avance III 400 MHz spectrometer, Milan, Italy), mass spectroscopy (MS) techniques, and optical rotation. Finally, it was elucidated as germichrysone. [𝛂]_D_ = +12; [𝛂]_546_ = +18; [𝛂]_436_ = +108 (c 0.15 g/100 ml, THF). ESI-MS: *m/z* 259.92 [M + H]^+^.

^1^H-NMR (400 MHz, MeOD) δ 6.88 (s, 1H), 6.78 (d, J = 2.4 Hz, 1H), 6.75 (dd, J = 2.3, 0.9 Hz, 1H), 4.32 (tt, J = 7.5, 3.7 Hz, 1H), 3.20 (dd, J = 15.6, 3.6 Hz, 1H), 2.97 (ddd, J = 5.1, 4.4, 2.4 Hz, 2H), and 2.73 (ddd, J = 17.2, 7.5, 0.9 Hz, 1H).

^13^C-NMR (MeOD): δ 203.5 (s), 158.9 (s), 145.1 (s), 140.5 (s), 136.1(s), 118.5(d), 113.0(d), 111.1 (s), 110.3 (s), 66.5 (d), 46.8 (t), 38.9 (t), and 22.2 (q).

### 2.4 Chiroptical methods

**VCD-IR**: measurements were carried out using a JASCO FVS 6000 FTIR instrument equipped with a ZnSe photo-elastic modulator (PEM), working at 50 kHz modulation, placed past a wire grid linear polarizer and with a lock-in amplifier after detection, and the latter was obtained with an MCT liquid N_2_-cooled diode for the regions 850–2000 cm^−1^. The tetrahydrofuran-d_8_ solution of germichrysone with 0.1 M concentration was recorded in a 200 mm BaF_2_ cell. A total of 5,000 scans were acquired, and a similar spectrum was taken for the solvent and subtracted out. **ORD**: measurements were performed with the use of a JASCO 815SE instrument with samples dissolved in tetrahydrofuran at 0.001 M concentration in 5-mm and 0.1-mm quartz cuvettes, in order to record the 350–500 nm and 200–350 nm ranges, respectively. A total of 10 scans per spectra were acquired. The ECD-UV spectra of the solvent were also recorded under the same conditions and subtracted thereafter from the sample ECD spectra. The UV spectra were obtained from the same apparatus. **ORD**: measurements were carried out with a JASCO P-2000 polarimeter. The tetrahydrofuran solution of germichrysone (0.15 g/100 ml) was recorded at three different wavelengths, 589 nm (Na lamp), 546 nm, and 435 nm (Hg lamp), with 10 measurements at each wavelength. **CPL**: the CPL and fluorescence spectra were recorded on a home-built apparatus (Castiglioni et al., 2010) in a 2-mm cell at the same concentration used for ECD-UV measurements. A total of 20 accumulation scans were employed. The excitation wavelength of 375 nm was obtained by using the commercial FP-8200 JASCO fluorimeter and brought to the cell through a water-filled optical fiber. **Calculations**: Density functional theory (DFT) and time-dependent density functional theory (TD-DFT) calculations were performed by use of the Gaussian16 program (Frisch et al., 2016) preceded by a conformational search of all possible conformers by the CREST program (Pracht et al., 2020) assuming (*R*)-configuration ((*R*)-AC) in setting up all calculations (Supplementary Material for details). The B3LYP/TZVP level was employed for DFT (optimizations and VCD-IR calculations), CAM-B3LYP/TZVP for ECD, and CAM-B3LYP/6-311++G (d,p) for ORD, which were used for TD-DFT calculations, both *in vacuo* and in PCM approximation with tetrahydrofuran as the solvent.

### 2.5 HPLC

HPLC-grade solvents were supplied by Honeywell (Seelze, Germany). The chromatography procedure was performed using a Thermo Finnigan (Japan) high-performance liquid chromatography–photodiode array system (HPLC-UV/PAD) equipped with a Surveyor Autosampler, a Surveyor Pump, and a Surveyor PDA Plus Detector. Experimental data were acquired and interpreted exploiting Xcalibur software. The stationary phase was represented by a reverse-phase column (Chromolith SpeedROD RP-18 endcapped column; 50 mm × 4.6 mm, ID 3 mm, macropore size 2 μm, mesopore size 13 nm, Merck, Darmstadt, Germany), while the mobile phase was water +0.1% HCOOH as solvent A and MeOH as solvent B in 15 min of analysis in total.

As shown in Table 3, the elution gradient and its timepoints are reported.

**TABLE 3:** Gradient elution of the developed HPLC method.

Time (min)	A (%)	B (%)
0	50	50
10	30	70
11	50	50
14	50	50

### 2.6 Cell culture

Human neuroblastoma SH-SY5Y cells were obtained from ATCC (Manassas, VA) and cultured in a humidified incubator at 37°C with 5% CO_2_. The SH-SY5Y cells were grown in Eagle’s minimum essential medium (EMEM) supplemented with 10% fetal bovine serum, 1% penicillin–streptomycin, l-glutamine (2 mM), non-essential amino acids (1 mM), and sodium pyruvate (1 mM).

RPMI 8226 human multiple myeloma cells (ATCC, Manassas, VA, United States) were cultured in RPMI 1640 medium (Euroclone, Pero, Italy) supplemented with 10% fetal bovine serum (FBS; GibcoR, Thermo Fisher Scientific, Lisbon, Portugal), 1% glutamine, and 1% penicillin and streptomycin (Euroclone, Pero, Italy). The cells were incubated at 37°C with 5% CO_2_ in a humidified incubator. (*R*)-ASME was dissolved in phosphate-buffered saline (PBS) at 1 g/ml concentration and then diluted directly into the culture medium to working concentrations.

### 2.7 Western blotting

SH-SY5Y cells were treated with 100 nM and 1 µM (*R*)-ASME for 1, 2, and 4 h and with 100 nM and 1 µM (*R*)-germichrysone for 4 h. Then, the cells were homogenized in a specific lysis buffer (Cell Signaling Technology, Denver, CO, United States). Proteins were diluted in 2× sodium dodecyl sulfate (SDS) protein gel loading solution, boiled for 5 min, separated onto 12% SDS-polyacrylamide gel electrophoresis (SDS-PAGE)), and processed following standard procedures. In each well, we loaded 40 µg of the total homogenate. The mouse monoclonal antibodies were diluted as follows: the anti-ELAVL4/HuD antibody (Sigma-Aldrich, Darmstadt, Germany SIGMA) at 1:1000; the anti-BDNF antibody (anti-brain-derived neurotrophic factor; Sigma-Aldrich) at 1:500; the anti-p62 antibody (Santa Cruz Biotech, Santa Cruz, California) at 1:1000; the anti-Ub antibody (anti-ubiquitin antibody; Santa Cruz Biotech) at 1:200; and the anti-α-tubulin antibody (Sigma-Aldrich) at 1:1000. The nitrocellulose membrane signals were detected via chemiluminescence (by using WesternBright^®^ ECL HRP substrate, Advansta, San Jose, CA, United States) by means of an Imager Amersham 680 detection system. Alpha-tubulin was used for data normalization. We used folic acid as a positive control, a compound that we previously demonstrated to be able to positively affect the protein expression of both HuD and BDNF (Marchesi et al., 2023). Statistical analysis was performed on the densitometric values obtained with the ImageJ image processing program.

### 2.8 Proteasome activity assay

#### 2.8.1 Enzymatic assay

The activity of (*R*)-ASME on the 20S proteasome, specifically its chymotrypsin-like catalytic site, was measured via a fluorogenic, non-radioactive and commercially available kit “20S Proteasome Assay Kit for Drug Discovery” (Enzo^®^ Life Sciences, Farmingdale, NY, United States, BML-AK740). Briefly, a solution of 20S proteasome at 10 μg/ml was prepared in Assay Buffer together with a 75 µM solution of the Suc-LLVY-AMC substrate and a 0.5 µM solution of epoxomicin in Assay Buffer. Several dilutions of (*R*)-ASME were chosen to be tested: 3 µM, 30 µM, 300 µM, 1 mM, 3 mM, 5 mM, and 10 mM. A 96-well plate was seeded as follows: blank (no proteasome, 40 µL of Assay Buffer and 10 µL of the fluorogenic substrate), control (no inhibitor, 30 µL of Assay Buffer, 10 µL of 20S proteasome solution, and 10 µL of the fluorogenic substrate), inhibitor (25 µL of Assay Buffer, 10 µL of 20S proteasome solution, 5 µL of epoxomicin, and 10 µL of the fluorogenic substrate), and test samples (25 µL of Assay Buffer, 10 µL of 20S proteasome solution, 5 µL of (*R*)-ASME dilutions, and 10 µL of the fluorogenic substrate). The analysis was performed in triplicate. After seeding, the plate was incubated at 30°C for 10 min to permit the inhibitor/enzyme interaction. After incubation, the substrate was added, starting the cleavage reaction. Excitation was set at 360 nm, and emission was measured at 460 nm with a FLUOstar Omega filter-based multi-mode microplate reader (BMG LABTECH Ltd., Aylesbury, UK). Data were recorded at 1-min intervals over 60 min of analysis and were subsequently analyzed following the manufacturer’s instructions: the data were plotted as arbitrary fluorescence unit/min (AFU/min), using a linear regression program. A line fitting considering only the timepoints in which the reaction is linear (from 0 to 10–15 min) was made and its slope calculated. The activity was obtained applying the following equation: 100-[(inhibitor slope/control slope)/x100]. Since (*R*)-ASME is a proteasome activator, the percentage was over 100%.

#### 2.8.2 Cellular assay for proteasome activity

RPMI 8226 cells were seeded at a density of 250 × 10^3^ cells/well in 6-well plates. After 24 h, the cells were treated with (*R*)-ASME (1–1000 mg/ml, i.e. 3 mM–3 M). At 24 h, the cells were collected, and the total protein extract was obtained. The lysis buffer used to obtain total protein extract was HEPES 5 mM pH 7.5, NaCl 150 mM, glycerol 10%, Triton ×100 1%, MgCl_2_ 1.5 mM, and EGTA 5 mM.

The protein content was quantified with the Bradford method. Forty micrograms of proteins, 10 µL of 10× proteasome buffer (HEPES pH 7.5 250 mM, EDTA pH 8.0 5 mM, NP-40 0.5%, and SDS 0.01%), and 10 µL of the proteasome substrate (N-succinyl-Leu-Leu-Val-Tyr-7-amido-4-methylcoumarin, 7.6 mg/ml) (Sigma-Aldrich, United States) were loaded in each well of a black 96-well plate. After 2 h at 37°C, fluorescence was quantified in a microplate reader (excitation 380 nm; emission 460 nm) (BMG LABTECH, Germany).

### 2.9 Statistical analysis

The GraphPad Prism statistical package (version 9, San Diego, CA, United States) was used for statistical analysis. The data were analyzed by analysis of variance (ANOVA) followed, when significant, by an appropriate *post hoc* comparison test, as detailed in the figure legends. Differences were considered statistically significant when *p*-value ≤0.05. The results are expressed as mean ± S.E.M.

## 3 Results

### 3.1 MCLib-2022 virtual screening

The computational approach pursued to identify dual ligands uses the SAFAN platform and docking experiments. SAFAN is a ligand-based method that evaluates the molecular similarity between the investigated molecules and the active compounds collected in the database. Briefly, the following steps were foreseen: molecular fragmentation, quantitative target affinity calculation, based on fragment and compound similarity, and WEKA machine learning approach. The output of the analysis is a list of potential targets, ranked in decreasing order (Supplementary Material). The compounds screened to identify new HuD binders belong to an in-house library (MCLib-2022) (Pagano et al., 2023). This library accounts for ca. 500 non-commercial small molecules synthesized or isolated from natural matrices by the authors’ research group. After computational analysis, nine main chemical clusters were identified within the library, and the representative general chemical structure was subjected to virtual screening. More information is available in the paper by Pagano et al. (2023). The SAFAN results highlighted that (*R*)-ASME, belonging to MCLib-2022, is a potential binder of HuD. Before proceeding to extract the selected metabolite to investigate its *in vitro* profile, we further *in silico* analyzed its interaction not only with HuD but also with the proteasome, in order to evaluate its multi-target potential.

### 3.2 Molecular recognition studies of (*R*)-ASME *versus* HuD and 20S proteasome *α*-rings

**FIGURE 1:**
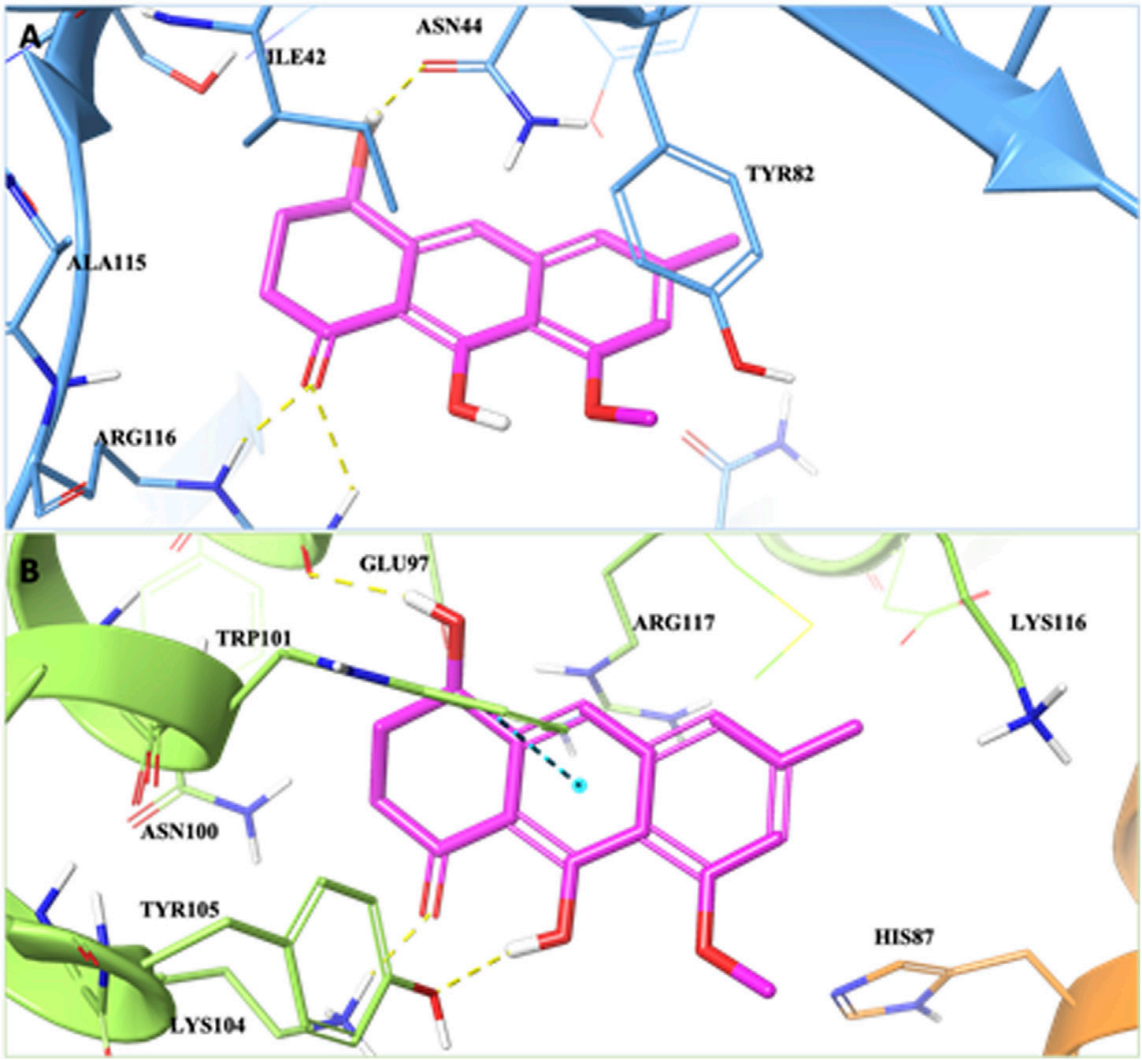
3D representation of the (*R*)-ASME complex in the **(A)** HuD binding pocket and **(B)** the α2/α6 intersubunit pocket of the 20S proteasome *α*-ring. The protein HuD protein is shown as blue, and α2/α6 subunits are shown as orange and green, respectively. The ligand is shown as magenta sticks. The residues involved in crucial contacts with the compounds are reported, respectively, as blue, orange, and green carbon sticks. Hydrogen bonds and π–π stacking interactions are reported, respectively, as yellow and cyan dashed lines.

Molecular recognition studies carried out on HuD highlighted that the ligand is well-accommodated in the protein pocket. In detail, (*R*)-ASME establishes three hydrogen bond interactions: one with Asn44 and two with Arg116 (Figure 1A). Moreover, the compound is engaged in several hydrophobic contacts with Ile42, Tyr45, Tyr82, Lys111, Arg123, Ile152, and Thr153.

In order to identify the putative binding mode of (*R*)-ASME *versus* the *α*-rings, molecular modeling studies were also carried out on the 20S proteasome. Due to the novelty of the target and the lack of information about the ligand-binding pocket on the *α*-rings, the SiteMap program was used to identify and characterize the putative ligand-binding sites on the 20S proteasome *α*-rings.

The molecular recognition results showed that the compound is accommodated in the α2/α6 intersubunit pocket, establishing hydrogen bond interactions with Glu97, Lys104, and Tyr105 and a π–π stacking interaction with Trp101 (Figure 1B). Moreover, (*R*)-ASME is engaged in several hydrophobic contacts with His87, Asn100, Tyr105, Lys116, and Arg117.

Thus, molecular recognition studies, carried out on HuD and *α*-rings of the 20S proteasome, revealed that the ligand is well-accommodated in both the binding sites.

### 3.3 Extraction and isolation optimization of (*R*)-ASME

To dispose of a suitable amount of (*R*)-ASME with adequate purity for the biological investigation, as a first step, we optimized the already described extraction procedure of *E. persicus* roots. First, an efficient analytical HPLC-UV/PAD method was set up. Considering the good resolution of the main peaks achieved in the previous work, we further optimized the method using the same column (Chromolith SpeedROD RP-18 endcapped column) and varied the gradient applied. As a result, the time of analysis was reduced from 30 to 15 min with no loss of resolution, thus containing the analysis costs and increasing the throughput.

**FIGURE 2:**
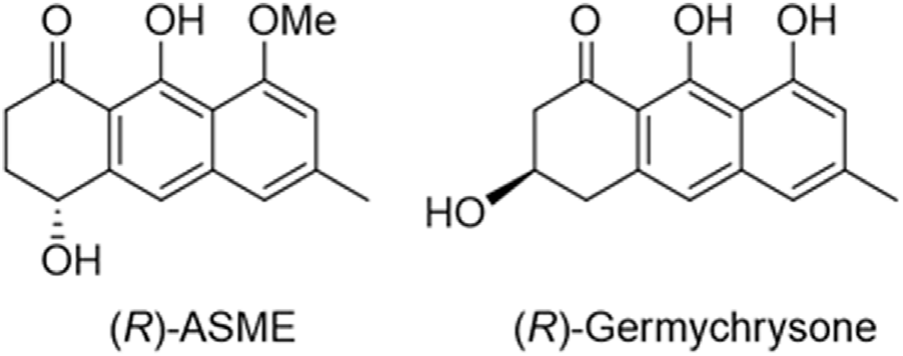
Chemical structure of (*R*)-ASME and (*R*)-germichrysone.

The next step was to optimize the extraction procedure by testing different protocols. Indeed, in our previous work, we developed a MASE protocol that required almost 1 hour, but never investigated the effect of time, temperature, and the heating source on the process. Specifically, the dried and powdered roots of *E. persicus* were subjected to either maceration (Mac) or microwave-assisted solvent extraction (MASE). The latter technique was selected for its several advantages such as higher efficiency and shorter extraction time. Furthermore, different extraction times (two or three cycles of 1 or 12 h for maceration and of 20 or 5 min for MASE) and temperatures (from room temperature to 120°C) were tested. All the raw extracts so obtained were next fractionated via liquid/liquid extraction using water/ethyl acetate. The organic fractions were then dried, evaporated under reduced pressure, and chromatographed to obtain two different pure metabolites. The best results were obtained performing two cycles (2 × 5 min) with solvent renewal at 60°C adopting an MASE approach (MASE_4). Thus, although maceration performed at 60°C is associated with a very high extraction yield, the advantages are not mirrored in the yield of pure metabolites. Indeed, the amount of ASME obtained following methods MASE_4 and Mac_4 is comparable, but microwaves offer a significant improvement in terms of time required (10 min vs. 13 h). This advantage can be explained considering the well-known higher efficiency related to microwave-assisted extraction (Martino et al., 2019; Cavalloro et al., 2021). Two metabolites have been thus isolated and properly characterized by MS and NMR spectroscopy: (*R*)-ASME and enantiomeric germichrysone, a secondary metabolite already identified in species belonging to the genus *Cassia* (Figure 2).

### 3.4 Assignment of absolute configuration of germichrysone

The absolute configuration (AC) of germichrysone, extracted from *Cassia torosa* Cavanilles, was determined from crystallographic data as (*R*), but in the same work, no optical rotation data were reported (Noguchi et al., 1978). Two years earlier, another work had measured negative optical rotation (-38, dioxane, c 0.52) for germichrysone extracted from the same plant (Takahashi et al., 1976). Conversely, the optical rotation of germichrysone extracted from *E. persicus* measured in tetrahydrofuran gave a positive value (Supplementary Table S3). Considering the opposite rotation of the compound extracted from *E. persicus* with respect to literature data and the low measured value, its AC was herein studied by means of full chiroptical spectroscopy encompassing also vibrational circular dichroism (VCD), electronic circular dichroism (ECD), and optical rotatory dispersion (ORD), together with their prediction via DFT and TD-DFT quantum mechanical calculations (Abbate et al., 2009; Nafie, 2011; Polavarapu, 2016; Mazzeo et al., 2017; Santoro et al., 2019). Moreover, since germichrysone is a fluorescent compound, its circularly polarized luminescence (CPL) activity was also measured.

**FIGURE 3:**
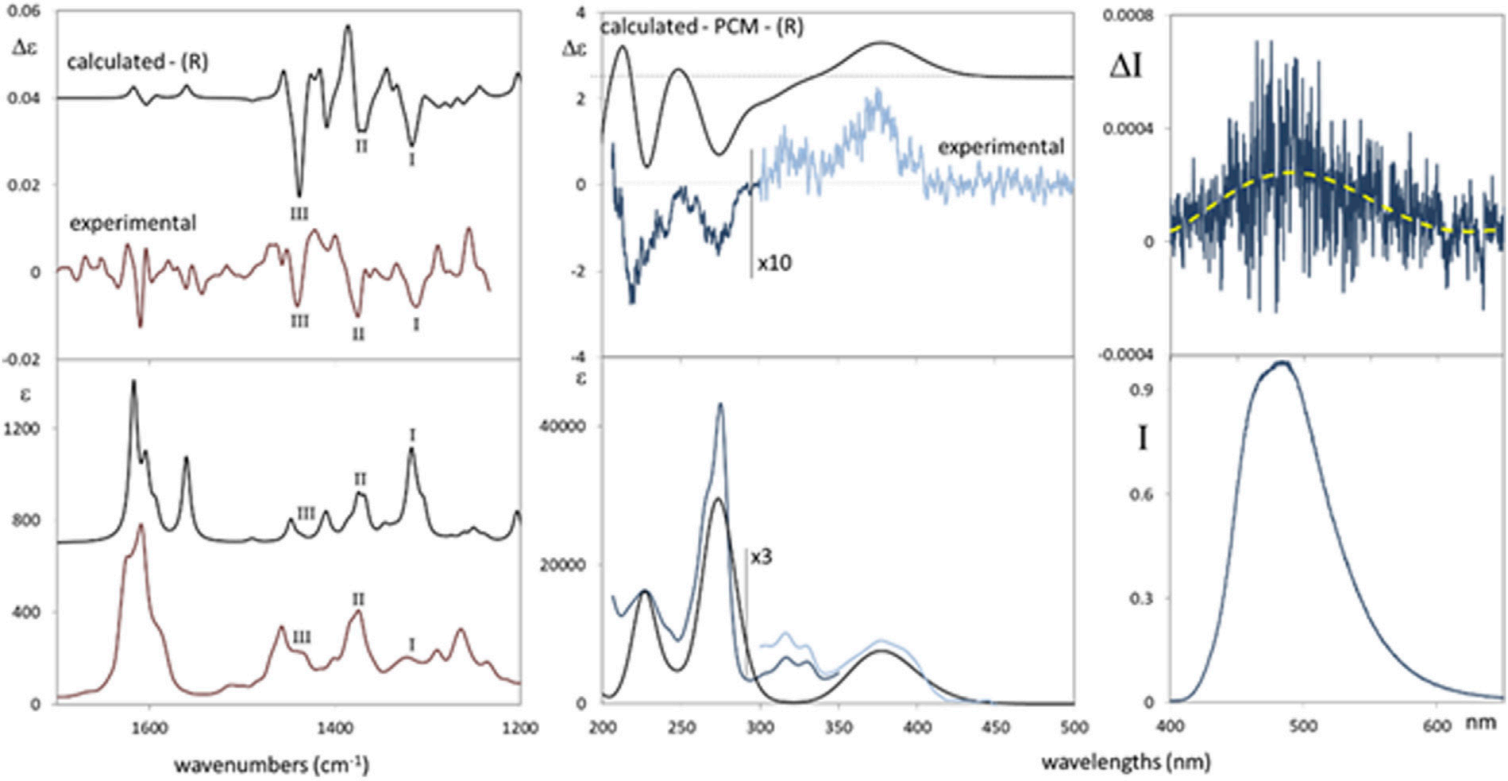
Comparison of the experimental and calculated VCD-IR (left panels) and ECD-UV (middle panels) spectra of germichrysone. For better comparison, light blue experimental spectra (500–300 nm range) were multiplied by a factor 10 for ECD (middle, top-right) and by a factor 3 for UV (middle, bottom right). Calculations, on (*R*)-germichrysone, were performed at the TD-DFT/CAM-B3LYP/TZVP level for ECD-UV and at DFT/B3LYP/TZVP for VCD-IR. The calculated ECD-UV spectra are red-shifted by 25 nm. The VCD-IR applied scaling factor is 0.97. Right panels: CPL (top right) and fluorescence (bottom right) spectra of germichrysone. Solvent: tetrahydrofuran. The excitation wavelength is 375 nm. The fluorescence band maximum is normalized to 1.

The ECD spectrum (Figure 3, middle) exhibits a weak positive band at ca. 375 nm, a second weak positive band allied to the UV feature centered at 325 nm, followed by a sequence of two negative bands at 275 nm and 220 nm. TD-DFT calculations satisfactorily are in agreement with experimental spectra in ECD band signs, relative magnitude, and position, thus supporting (*R*)-AC. In Supplementary Figures S1, S2, we report all conformers’ calculated structures and corresponding ECD spectra, respectively. Six most stable conformers were found (Supplementary Table S2): three with the OH group in the cyclohexanone ring in equatorial conformation and three in axial conformation, corresponding to the three rotameric states of the aliphatic hydroxyl group. Equatorial and axial conformers contribute differently to averaged ECD in an almost enantiomeric fashion, making the calculation of ECD to be dramatically sensitive to conformer relative population evaluation. For this reason, also ORD and VCD-IR spectra have been considered. OR has been measured at three different wavelengths (Supplementary Table S3): +12 at 589 nm, +18 at 546 nm, and +108 at 436 nm. Quantum mechanics calculations of OR values (assuming (R)-AC) give a good prediction of sign and order of magnitude, thus confirming (R)-AC, as reported in the ECD-UV case.

Complementary to ECD data, it is also interesting to consider the CPL and fluorescence spectra (Figure 3, right). A structured fluorescence band is centered at 480 nm (showing a ca. 100 nm Stokes shift with respect to the low-energy UV band set at 375 nm); the CPL spectrum shows a positive band matching the sign of the low-energy experimental ECD band (Figure 3, middle); this is the most standard behavior observed for organic compounds, in which the ground and excited states retain a similar structure (Longhi et al., 2016). The dissymmetry ratio g_lum_ (defined as Δ/I) is quite low (ca. 4∙10^−4^) but larger than its absorption counterpart g_abs_ (evaluated as Δε/ε for the lowest energy transition CD band), which is ca. 6∙10^−5^. It is also worth noting that the large Stokes shift experienced suggests an excited state intramolecular proton transfer process, which was recently investigated through fluorescence and CPL in natural compounds (Mazzeo et al., 2023).

Finally, to provide further evidence for the assigned AC, VCD and IR spectra were also measured and calculated (Figure 3, left). The main experimental VCD and IR features (IIII) are well-predicted. However, the experimental IR band I, at ca. 1315 cm^−1^, is overestimated by the calculation, although it is well-predicted in VCD as a negative band. Experimental bands II and III, at ca. 1370 cm^−1^ and 1430 cm^−1^, respectively, also provided negative features in the experimental VCD spectrum and are satisfactorily predicted by the calculation. Taken together, obtained VCD-IR results support (*R*)-AC for germichrysone, in agreement with ECD and ORD investigations.

### 3.5 *In vitro* assessment

#### 3.5.1 Ability to bind HuD

The effect of 100 nM and 1 µM (*R*)-ASME on HuD and BDNF protein expression was evaluated at different timeframes, namely, 1, 2, and 4 h. The same investigation was carried out for (*R*)-germichrysone at the same concentrations (1 μM and 100 nM) but only at 4 h. The concentrations and times of exposure were chosen according to our previous published paper (Marchesi et al., 2023) and to our own experience as being optimal for BDNF transcriptional activity, mRNA half-life (t1/2 132 ± 30 min), and protein expression (see also Castrén et al., 1998).

**FIGURE 4:**
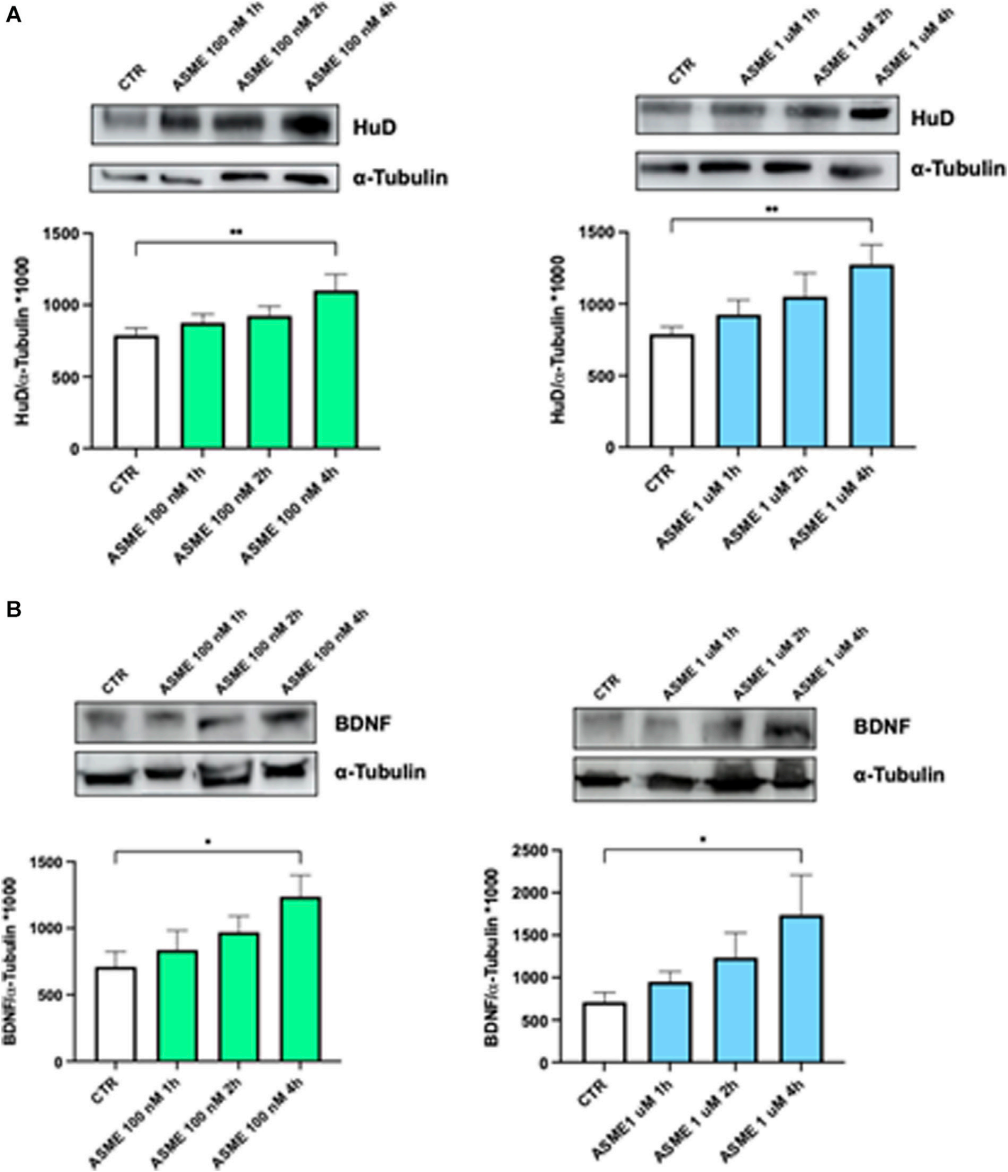
HuD and BDNF expression following 1, 2, and 4 h of (*R*)-ASME treatment. **(A, B)** Densitometric analysis of HuD **(A)** and BDNF **(B)** proteins and the respective *α*-tubulin in the total homogenates of SH-SY5Y cells following exposure to solvent (0.1% of DMSO; CTR) or (*R*)-ASME (ASME) 100 nM (left) and 1 µM (right) for 1 h, 2 h, and 4 h. The results are expressed as mean gray level ratios (mean ± S.E.M.) of HuD/α-tubulin **(A)** and BDNF/α-tubulin **(B)** ×1000. *p < 0.05, **p < 0.01, Dunnett’s multiple comparisons test, n = 6 independent samples.

The results obtained show that (*R*)-ASME is able to significantly increase HuD (Figure 4A) and BDNF (Figure 4B) levels at both concentrations following 4-h incubation. Conversely, (*R*)-germichrysone negatively affects the expression of both HuD and BDNF proteins at both tested concentrations after 4-h exposure (Supplementary Figure S4).

#### 3.5.2 Proteasome enzymatic activity

**FIGURE 5:**
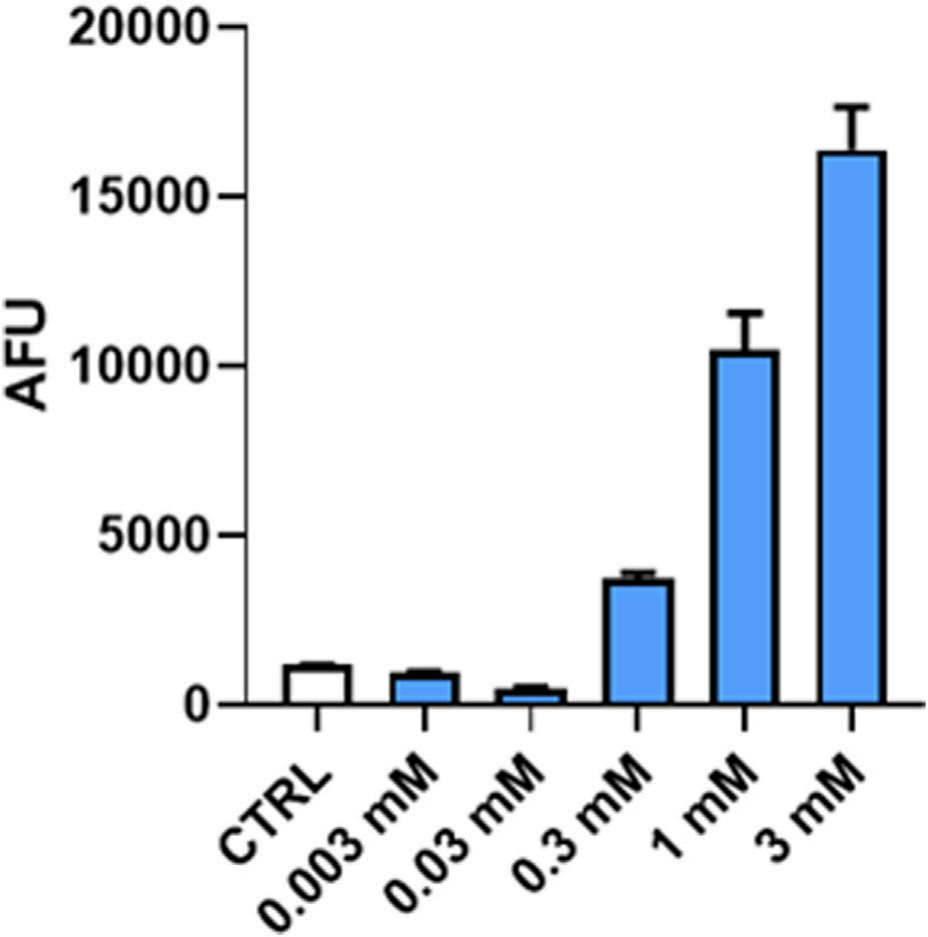
Plot of arbitrary fluorescence units (AFUs) against (*R*)-ASME concentration [mM] at a 10-min time point. The results are expressed as AFU mean (mean ± S.E.M.).

The direct interaction of (*R*)-ASME with the chymotrypsin-like protease active site of the 20S proteasome was evaluated by exploiting the commercially available 20S Proteasome Assay Kit for drug discovery. (*R*)-ASME is able to directly activate the 20S proteasome in a dose-dependent manner. In particular, (*R*)-ASME enhances the activity 6-fold at 300 µM, reaching a plateau at 3 mM with a 26-fold increase (Figure 5).

#### 3.5.3 Proteasome activity in RPMI cells

**FIGURE 6:**
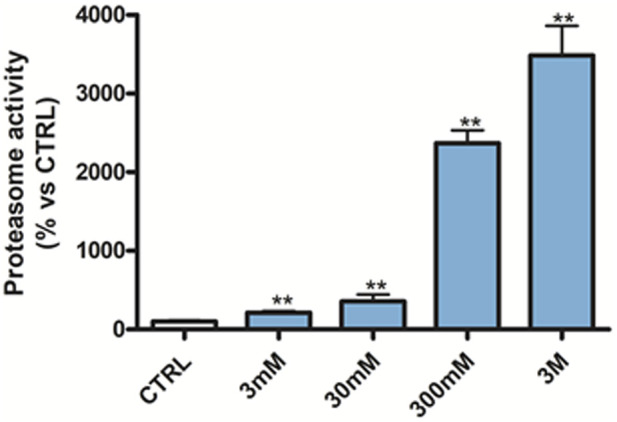
Proteasome activity in RPMI 8226 cells following 24 h of 3 mM–3 M (*R*)-ASME treatment. The results are expressed as mean percentage (mean ± S.E.M.) compared to untreated CTRL. **p < 0.01, Dunnett’s multiple comparisons test, n = 3 independent experiments.

The effect of (*R*)-ASME on the proteasome was initially tested on RPMI cells. (*R*)-ASME was able to enhance proteasome activity in a dose-dependent manner, doubling the activity at 3 mM and tripling it at 30 mM (Figure 6).

#### 3.5.4 Proteasome activity in SH-SY5Y cells

**FIGURE 7:**
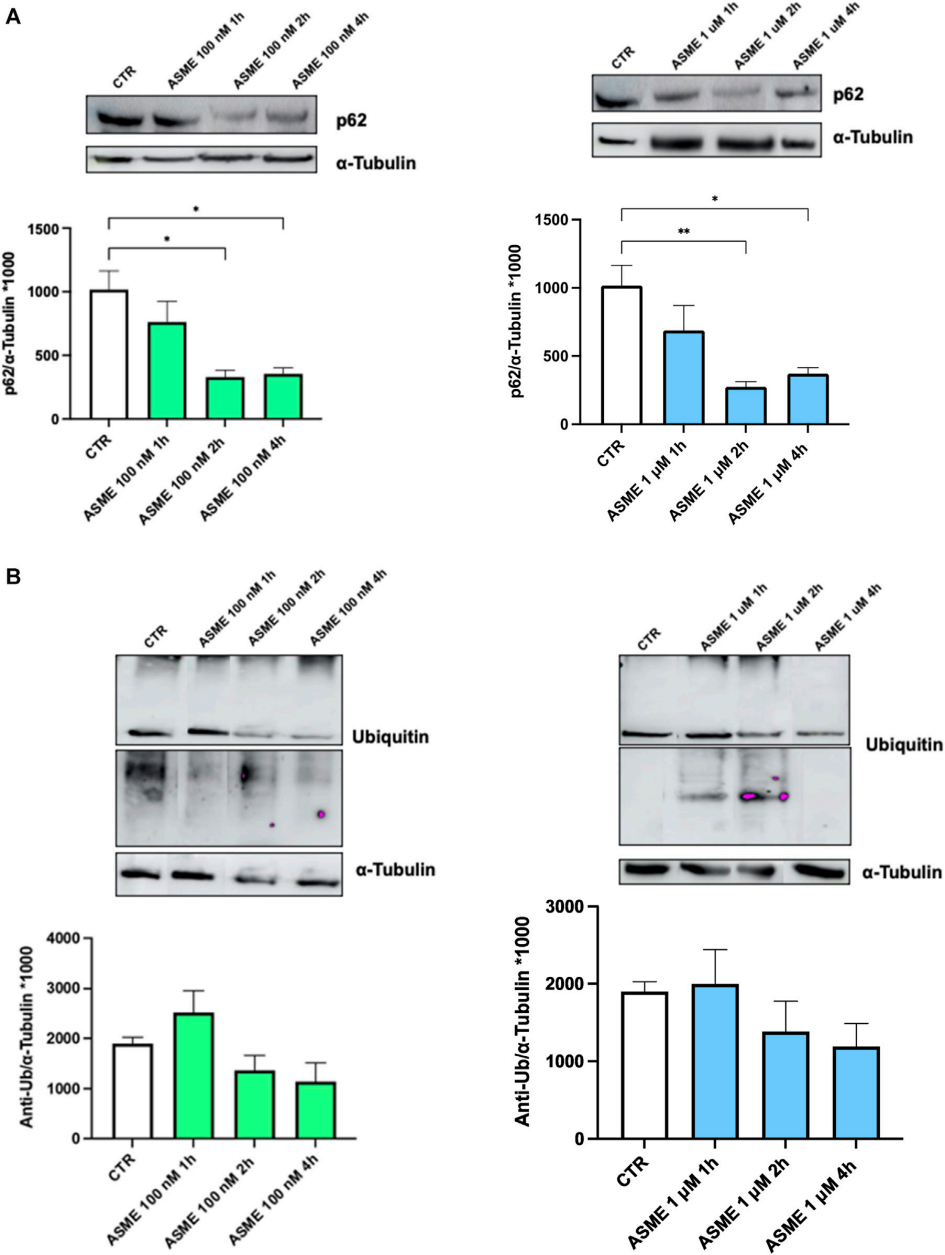
P62 and ubiquitin expression following 1, 2, and 4 h of (*R*)-ASME treatment. **(A, B)** Densitometric analysis of p62 **(A)** and ubiquitin (anti-Ub; **(B)** proteins and the respective *α*-tubulin in the total homogenates of SH-SY5Y cells following exposure to the solvent (0.1% of DMSO; CTR) or 100 nM (left) and 1 µM (right) (*R*)-ASME (ASME) for 1 h, 2 h, and 4 h. The results are expressed as mean gray level ratios (mean ± S.E.M.) of p62/α-tubulin **(A)** and ubiquitin/α-tubulin **(B)** ×1000. *p < 0.05, **p < 0.01, Dunnett’s multiple comparisons test, n = 6 independent samples.

The effect of 100 nM and 1 µM (*R*)-ASME on p62 expression was evaluated at different timeframes. As shown in Figure 7 (panel A), (*R*)-ASME was able to significantly reduce the level of p62, which is an indicator of protein breakdown via the proteasome, after 4 h at both concentrations. Notably, the decrease of p62 is an index indicating that the degradation phase was successful, while its increase reveals that the degradation has not occurred. The levels of the ubiquitination of folded/unfolded proteins that will undergo degradation and the effect of (*R*)-ASME on this process were also investigated at the same timeframes and concentrations. As shown in Figure 7 (panel B), (*R*)-ASME is able to induce a trend toward a decrease, thus indicating that the degradation phase was successful.

## 4 Discussion

Despite the great efforts of the scientific community to discover new effective drugs against neurodegenerative diseases, there is still a lack of effective cures for these pathologies. Therefore, there is an urgent need for molecules acting through novel and not fully explored mechanisms of action, thus enabling a significant breakthrough in this field. Among the recently explored targets, here we focused on the HuD protein and the proteasome system, which may represent valuable candidate targets for the treatment of several relevant neurodegenerative diseases. HuD is still poorly investigated from a medicinal chemistry perspective, and only a few molecules able to bind this target have been identified by now (Ambrosio et al., 2021; Silvestri et al., 2022; Zhang et al., 2023). Among these, folic acid (a vitamin widely used in the prevention of some neurodegenerative diseases) is worth mentioning as a modulator of HuD activity (Ambrosio et al., 2021; Marchesi et al., 2023).

To find new actives potentially able to interact with HuD, we screened the in-house library MCLib-2022, which groups all the molecules synthesized or isolated from natural matrices by our research group along 30 years of medicinal chemistry research activity and thus covering a wide chemical space. Computational studies showed that (*R*)-aloesaponol-III-8-methyl ether [(*R*)-ASME] has the potential to bind the HuD protein and to interact with the ubiquitin–proteasome system, a potential target useful in the development of agents to counteract neurodegenerative diseases. Briefly, the secondary metabolite (*R*)-ASME has been identified from *Eremurus persicus* Jaub & Spach) Boiss by our group in a drug discovery campaign (Rossi et al., 2017). This plant is well-known by the local people for its beneficial effects on human health, and traditional medicine recognizes different activities depending on the part of the plant considered (Beiranvand and Beiranvand, 2022). Of note, the neuroprotective activity of *E*. *persicus* extracts has never been documented before, thus representing a new potential application.

Prior to the biological investigation, we performed *in silico* analysis to deeply investigate its interaction with the above-mentioned targets. Docking results confirmed the multi-target potential profile of (*R*)-ASME. In fact, we highlighted that the compound is well-accommodated in both the HuD binding site and in the α2/α6 intersubunit pocket of the 20S proteasome. The direct interaction with the selected target is an important feature of (*R*)-ASME, as non-specific interactions are often associated with several side effects and poor efficacy.

To investigate its potential effect on HuD and the proteasome system and to dispose of a suitable amount with adequate purity for the biological investigation, we optimized the previously described extraction procedure of *E. persicus* roots. The new protocol allowed us to shorten both the extraction and analysis time and to simplify the fractionation protocol. Furthermore, we isolated and identified the structurally related germichrysone together with (*R*)-ASME. We then assigned the (*R*)-AC to germichrysone by means of full chiroptical spectroscopy investigation including VCD, ECD, and ORD, together with their prediction by DFT and TD-DFT quantum mechanical calculations, with all these methods tested as the most reliable ways to assign the AC to compounds in solution (Abbate et al., 2009; Nafie, 2011; Polavarapu, 2016; Mazzeo et al., 2017; Santoro et al., 2019). Moreover, since germichrysone is a fluorescent compound, we also measured and detected its CPL activity. Notably, this is the first time (*R*)-germichrysone has been isolated from the genus *Eremurus*.

The *in vitro* investigation was carried out on both compounds, (*R*)-ASME and (*R*)-germichrysone. First, their effect on the HuD protein levels was evaluated, and then the investigation was extended to BDNF, considering that the BDNF transcript is a HuD target and that its corresponding protein is implicated in several neuronal diseases and involved in the regulation of neuronal development, survival, and function. Consistent with SAFAN-ISP prediction and docking evaluation, (*R*)-ASME exposure leads to a significant increase in the HuD protein amount. Moreover, once bound by (*R*)-ASME, the HuD protein is activated, exerting a positive effect on its downstream target BDNF, and likely on itself, in line with our previously published data (Marchesi et al., 2023). Taken together, these findings strongly suggest that (*R*)-ASME is able to effectively regulate the HuD/BDNF cascade. This outcome is of particular interest, as HuD is still a poorly investigated target, and the number of its already identified binders is limited. Considering this, its identification could represent a step ahead in the study of the structure–activity relationship of HuD binders. Conversely, (*R*)-germichrysone, although structurally related to (*R*)-ASME, is not active.

Regarding the effect of (*R*)-ASME on the proteasome system, we demonstrated that this compound directly activates the 20S proteasome. Of note, unlike the 26S proteasome, which degrades ubiquitinylated proteins, the 20S proteasome is able to degrade only disordered proteins, and many of its enhancers have already been identified in George and Tepe (2021). The chemical structure of these molecules may vary, but no enhancers structurally related to (*R*)-ASME have been identified yet. Further results obtained in RPMI (a cell line in which the proteasome activity is highly dysregulated) and in SH-SY5Y cells clearly demonstrate the ability of (*R*)-ASME to operate as an activator of the proteasome complex, as evidenced by the decreasing of both the expression of both p62 (an indicator of degradation) and ubiquitin (a marker of proteins undergoing degradation).

The dual nature of (*R*)-ASME could make this molecule suitable for fighting multifactorial diseases such as neurodegenerative diseases.

## 5 Conclusion

In the present work, we have identified (*R*)-ASME as a multi-target ligand, potentially useful in counteracting neurodegenerative diseases, characterized by closely related mechanisms of action: the ability to act on the HuD/BDNF cascade and to activate the ubiquitin–proteasome system, two orthogonal mechanisms that may act in an additive/synergistic manner. The results of the molecular recognition studies confirm those obtained by preliminary virtual screening and, more importantly, consistent with those obtained by virtual screening. Overall, these results suggest an important prospective use of (*R*)-ASME in neurodegenerative diseases, particularly those characterized by an altered protein degradation system, such as Alzheimer’s disease. Furthermore, the results presented here lay the foundation for the identification of novel hit/lead compounds with a multi-target profile.

These results also pave the way for the development of new precision medicine. Thus, this approach is still poorly applicable in the neurodegenerative field, as it usually focuses on a single-target perspective, whereas a multi-target approach may be required for neurodegenerative diseases with complex molecular pathways.

## Data Availability

The original contributions presented in the study are included in the article/Supplementary Material; further inquiries can be directed to the corresponding authors.
