# Supplementary material for: Neurodegeneration: can metabolites from Eremurus persicus help?
Source: Front Pharmacol. 2024 Feb 5;15:1309766. doi: 10.3389/fphar.2024.1309766 (PMC10873958; doi:10.3389/fphar.2024.1309766)
Supplement: Supplementary file 1 [file DataSheet1.docx]

Supplementary Material

# SAFAN results

The output of SAFAN analysis is a list of potential targets, ranked in a decreasing order. Here below results related to (*R*)-ASME are reported.

| **Target: Mnemonic Name** | **Target: Gene Name** | **pK** | **Average Similarity with SAFAN-ISP fragments** | **Average Similarity with SAFAN-ISP compounds** | **Predicted Absolute Error** |
| --- | --- | --- | --- | --- | --- |
| ELAV1 | ELAVL1 | 7.06 | ? | 0.92 | 0.53 |
| O97447_nomam |  | 5.9 | ? | 0.711 | 0.66 |
| GLD1_nomam | GLD1 | 5.53 | ? | 0.711 | 0.66 |
| HD | HTT | 5.5 | ? | 0.711 | 0.66 |
| REV_nomam | REV | 4.79 | ? | 0.711 | 0.66 |
| TAU | MAPT | 4.619 | ? | 0.701 | 0.66 |
| KDM4E | KDM4E | 4.519 | ? | 0.699 | 0.71 |
| KMT2A | KMT2A | 4.459 | ? | 0.696 | 0.71 |
| NPSR1 | NPSR1 | 4.7 | ? | 0.668 | 0.71 |
| NPC1 | NPC1 | 5.469 | 0.654 | 0.647 | 0.71 |
| FRIL | FTL | 4.869 | ? | 0.643 | 0.71 |
| DYR1A | DYRK1A | 5.54 | ? | 0.641 | 0.71 |
| APEX1 | APEX1 | 4.85 | ? | 0.641 | 0.71 |
| Q194T2_nomam |  | 5.177 | 0.654 | 0.638 | 0.71 |
| DPOLB | POLB | 4.414 | ? | 0.625 | 0.71 |
| RORG | RORC | 5.257 | 0.654 | 0.617 | 0.71 |
| ACES_nomam | ACHE | 6.629 | 0.647 | 0.579 | 0.81 |
| AL1A1 | ALDH1A1 | 4.948 | ? | 0.575 | 0.81 |
| AMPC_nomam | AMPC | 4.937 | 0.654 | 0.573 | 0.81 |

**Supplementary Table 1.** Example of SAFAN report related to (*R*)-ASME

# Absolute configuration assignment of (*R*)-germichrysone

In Figure 3 of the main text, we reported experimental VCD-IR and ECD-UV spectra of germichrysone compared with their calculated counterparts, assuming (*R*)-AC at carbon 3. In the following we summarize some computational details.

**Results from conformational analysis.**


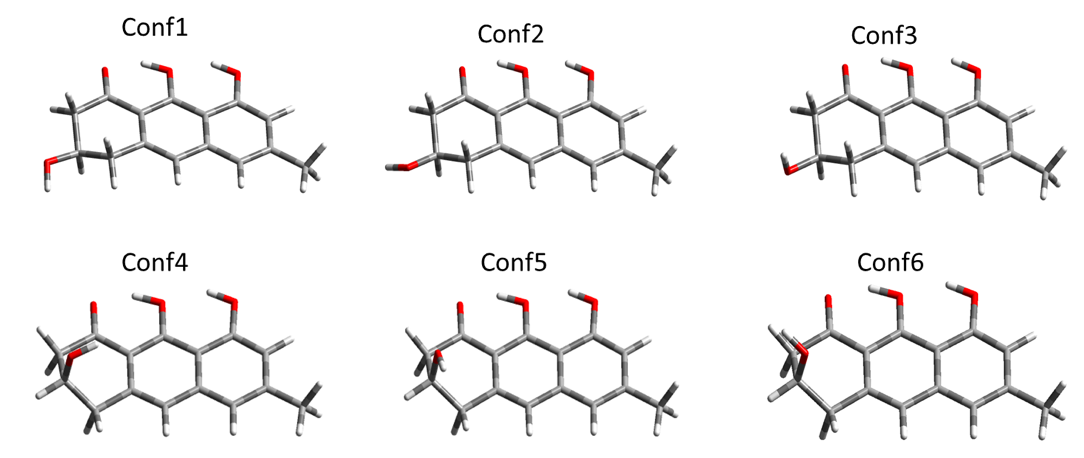


**Supplementary Figure 1.** Calculated geometries of (*R*)-germichrysone at DFT/B3LYP/TZVP level of theory.

| **Conformers** | **ΔG_gas_** | **%pop** | **ΔG_THF_** | **%pop** |
| --- | --- | --- | --- | --- |
| 1_eq_ | 0.000 | 30.0 | 0.087 | 24.4 |
| 2_eq_ | 0.004 | 29.8 | 0.000 | 28.3 |
| 3_eq_ | 0.461 | 13.8 | 0.494 | 14.0 |
| 4_ax_ | 0.545 | 12.0 | 0.813 | 7.2 |
| 5_ax_ | 0.754 | 8.4 | 0.411 | 14.1 |
| 6_ax_ | 0.952 | 6.0 | 0.503 | 12.1 |

**Supplementary Table 2.** Conformers, free energies (in kcal/mol) calculated *in vacuo* and in THF/PCM approximation, and relative population percentages at DFT/B3LYP/TZVP level of theory for (*R*)-germichrysone. Subscripts *eq* and *ax* refer to equatorial or axial conformation of hydroxyl group on carbon 3.

**ECD spectra calculated for the populated conformers.**


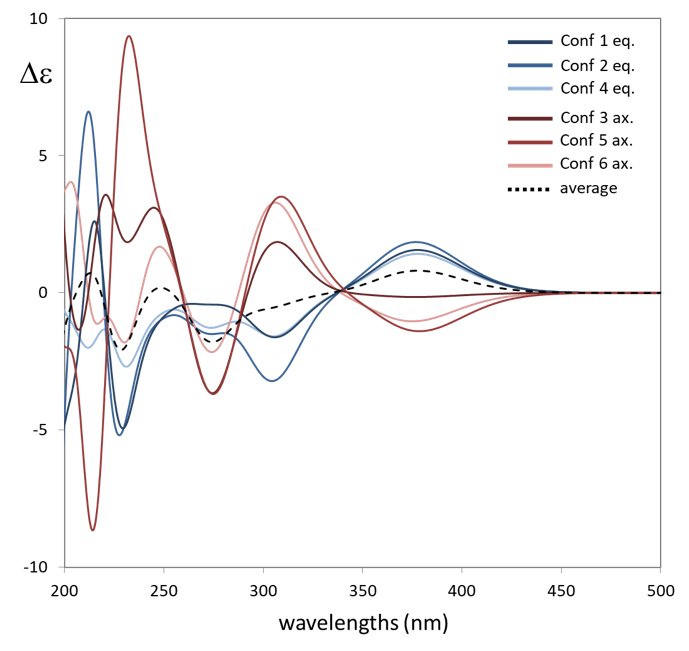
The three equatorial conformers contribute to ECD experimental positive band at 375 nm (HOMO-LUMO transition) and to negative band at ca. 220 nm. OH-axial conformers contribute instead to negative band at 275 nm. The overall contribution of equatorial conformers is ca. 75% according to *in vacuo* calculations and is ca. 66% in polarizable continuum model (PCM) approximation (see Table SI-1). The averaged ECD calculated spectrum shows a good matching (in either PCM or *in vacuo* conditions) with the experimental one, unambiguously supporting (*R*) AC of germichrysone. For sake of simplicity only PCM results have been reported in Figure S2.

**Supplementary Figure 2.** Calculated ECD spectra of (*R*)-germichrysone single conformers (solid lines) and average (dashed line) at CAM-B3LYP/TZVP level. Calculated ECD spectra are red shifted by 25 nm.

**OR calculations.**

Quantum-mechanics calculations of OR values are reported in Table S2.

| wavelength | **Optical Rotation** | | | | | | | |
| --- | --- | --- | --- | --- | --- | --- | --- | --- |
| **nm** | ***exp*** | **Conf1**  ***eq*** | **Conf2**  ***eq*** | **Conf3**  ***eq*** | **Conf4**  ***ax*** | **Conf5**  ***ax*** | **Conf6**  ***ax*** | **avg** |
| 589 | +12 | +8 | +49 | +46 | -33 | -45 | +29 | +17 |
| 546 | +18 | +17 | +66 | +63 | -47 | -59 | +34 | +25 |
| 436 | +108 | +143 | +220 | +215 | -209 | -173 | +33 | +101 |

**Supplementary Table 3.** Experimental (exp) and calculated (considering (*R*) as AC) single conformers and averaged (avg) specific optical rotation of germichrysone, measured in THF solvent at c 0.15 g/100 mL, at three different wavelengths. ORD calculations were performed at TD-DFT/CAM-B3LYP/6-311++G(d,p) level of theory.

Positive ORD trend is found in 80% of overall population: all conformers in equatorial OH conformation contributed to positive OR values while axial ones to negative values except for conformer 6. Calculated weighed ORD positive trend is also not affected by use of PCM or *in vacuo* condition then supporting (*R*)-AC for germichrysone as reported in ECD-UV case.

**VCD spectra calculated for the populated conformers.**


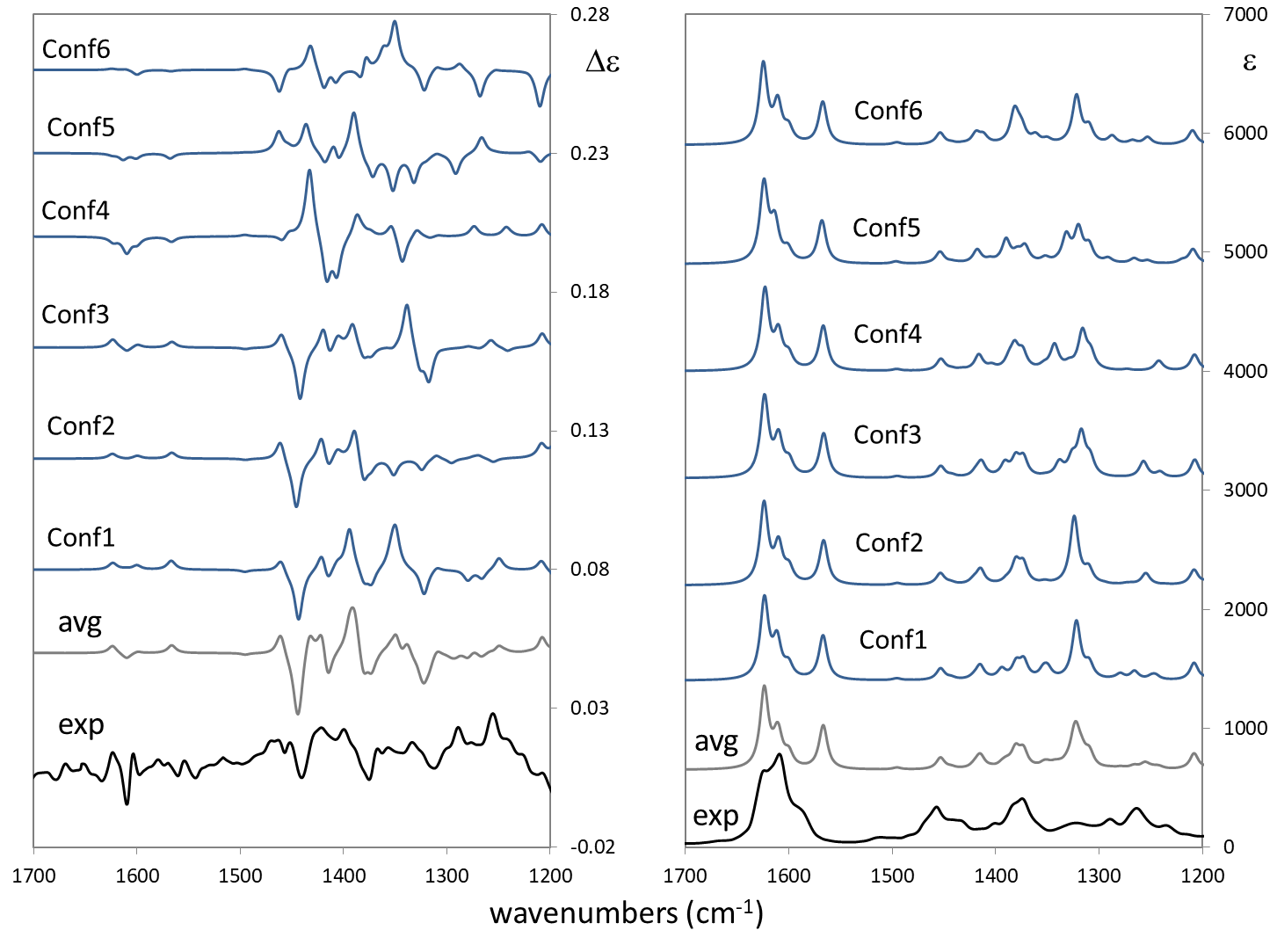


**Supplementary Figure 3.** Calculated VCD (left) and IR (right) spectra of germichrysone and (*R*)-AC conformers at B3LYP/TZVP level.

In Figure S3 we report experimental, calculated averaged and single conformers VCD-IR spectra. The main experimental VCD and IR features (I-III) are well predicted. II and III are associated to CH_3_, aromatic OH and C*-H bendings (1370 cm^-1^), CH_2_ scissoring bendings, C*-H and aliphatic OH bendings (1430 cm^-1^). Three experimental IR bands are also present at higher wavenumbers: 1585 cm^-1^ (as a shoulder), 1610 cm^-1^ and 1620 cm^-1^ giving rise to a weak triplet (+,-,+) in VCD.

# In vitro assessment on (*R*)-Germichrysone: ability to bind HuD

The effect of 100 nM and 1 µM (*R*)-germichrysone on HuD protein expression was evaluated at 4 hours. As can be noticed in Figure S4, the analyzed metabolite didn’t affect in a positive manner the expression of both HuD and BDNF.


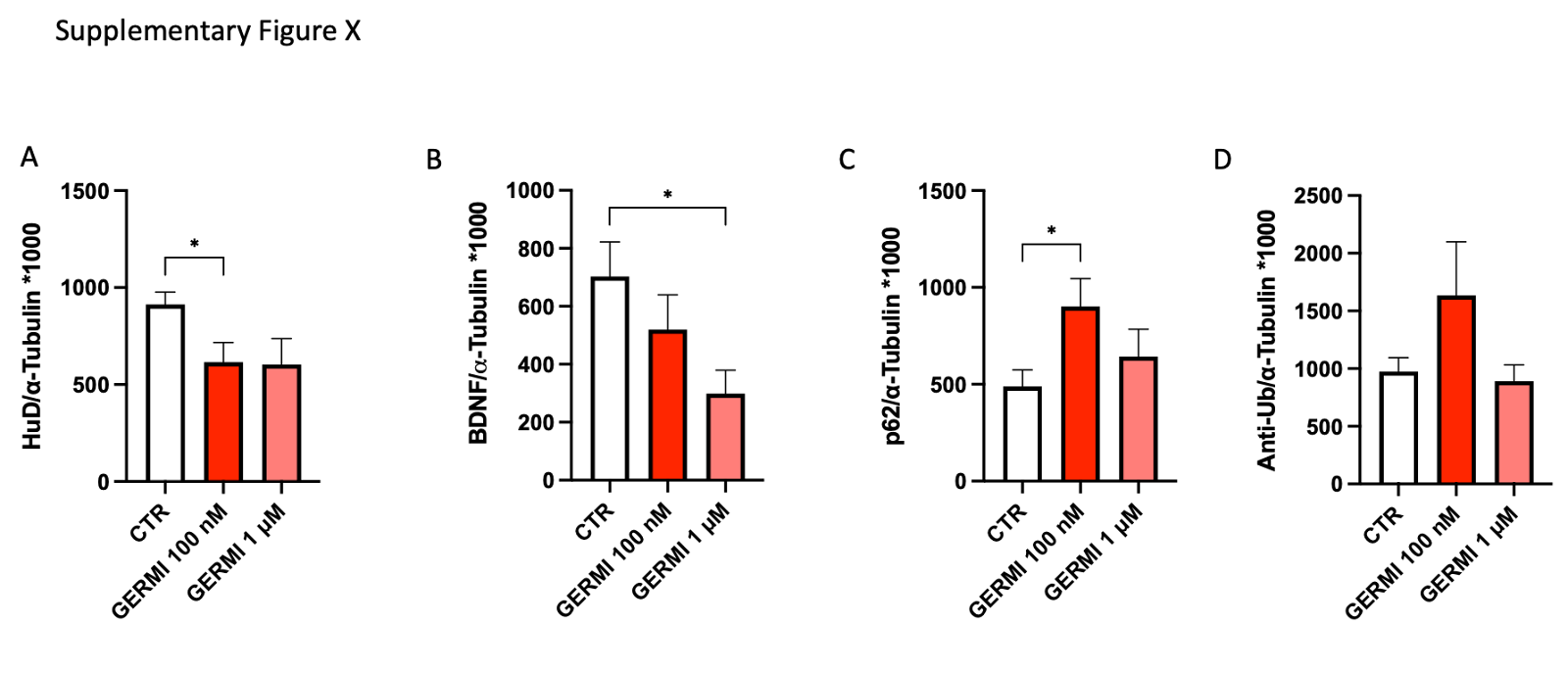


**Supplementary Figure 4.** Densitometric analysis of HuD (A), BDNF (B), p62 (C) and ant-ubiquitin (D) proteins and the respective a-tubulin in the total homogenates of SH-SY5Y cells following exposure to solvent 0.1% of DMSO (CTR) or (*R*)- germichrysone (GERMI) at 4h. Results are expressed as mean grey levels ratios (mean ± S.E.M.) of p62/a-tubulin (A) and ant-ubiquitin (Anti-Ub) / a-tubulin (B) ×1000. *p < 0.05, **p < 0.01, Dunnett’'s multiple comparisons test, n=6  independent samples.
